# Supplementary figures and images for: Ocular manifestations in a cohort of patients with mpox in the Democratic Republic of the Congo 2007–2011
Source: ASM Case Rep. 2026 Feb 11;2(2):e00171-25. doi: 10.1128/asmcr.00171-25 (PMC12955382; doi:10.1128/asmcr.00171-25)

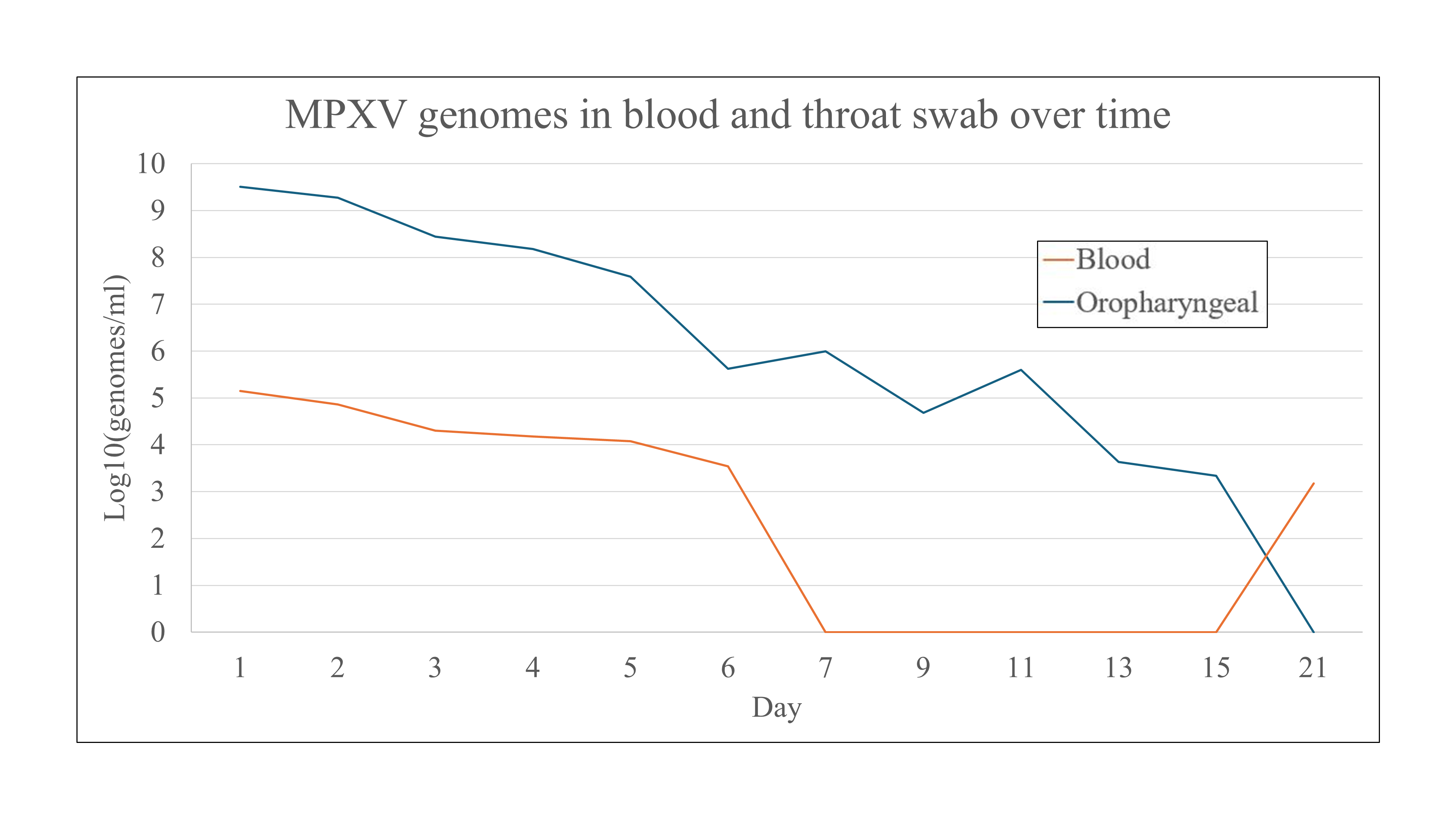

Supplement: Figure S1 — Monkeypox genome concentration in blood and oropharyngeal swab over time in the patient described. [file asmcr.00171-25-s0001.tif]
